# Machine‐learning model for the prediction of acute orthostatic hypotension after levodopa administration

**DOI:** 10.1111/cns.14575

**Published:** 2024-03-11

**Authors:** Zhu Liu, Shinuan Lin, Junhong Zhou, Xuemei Wang, Zhan Wang, Yaqin Yang, Huizi Ma, Zhonglue Chen, Kang Ren, Lingyu Wu, Haimei Zhuang, Yun Ling, Tao Feng

**Affiliations:** ^1^ Department of Neurology, Center for Movement Disorders, Beijing Tiantan Hospital Capital Medical University Beijing China; ^2^ China National Clinical Research Center for Neurological Diseases Beijing China; ^3^ GYENNO SCIENCE CO., LTD. Shenzhen China; ^4^ HUST – GYENNO CNS Intelligent Digital Medicine Technology Center Wuhan China; ^5^ Hinda and Arthur Marcus Institute for Aging Research Hebrew SeniorLife Roslindale Massachusetts USA; ^6^ Harvard Medical School Boston Massachusetts USA

**Keywords:** leave‐one‐out cross‐validation, levodopa, levodopa challenge test, orthostatic hypotension, Parkinson's disease, predictive model, random forest, supine‐to‐standing test

## Abstract

**Background:**

Levodopa could induce orthostatic hypotension (OH) in Parkinson's disease (PD) patients. Accurate prediction of acute OH post levodopa (AOHPL) is important for rational drug use in PD patients. Here, we develop and validate a prediction model of AOHPL to facilitate physicians in identifying patients at higher probability of developing AOHPL.

**Methods:**

The study involved 497 PD inpatients who underwent a levodopa challenge test (LCT) and the supine‐to‐standing test (STS) four times during LCT. Patients were divided into two groups based on whether OH occurred during levodopa effectiveness (AOHPL) or not (non‐AOHPL). The dataset was randomly split into training (80%) and independent test data (20%). Several models were trained and compared for discrimination between AOHPL and non‐AOHPL. Final model was evaluated on independent test data. Shapley additive explanations (SHAP) values were employed to reveal how variables explain specific predictions for given observations in the independent test data.

**Results:**

We included 180 PD patients without AOHPL and 194 PD patients with AOHPL to develop and validate predictive models. Random Forest was selected as our final model as its leave‐one‐out cross validation performance [AUC_ROC 0.776, accuracy 73.6%, sensitivity 71.6%, specificity 75.7%] outperformed other models. The most crucial features in this predictive model were the maximal SBP drop and DBP drop of STS before medication (ΔSBP/ΔDBP). We achieved a prediction accuracy of 72% on independent test data. ΔSBP, ΔDBP, and standing mean artery pressure were the top three variables that contributed most to the predictions across all individual observations in the independent test data.

**Conclusions:**

The validated classifier could serve as a valuable tool for clinicians, offering the probability of a patient developing AOHPL at an early stage. This supports clinical decision‐making, potentially enhancing the quality of life for PD patients.

## Graphical abstract

We achieved a prediction accuracy of 72% on test data for prediction of acute orthostatic hypotension post levodopa (AOHPL) of Parkinson's disease patients. This validated classifier could help clinicians obtain the probability of developing AOHPL of a patient early, providing a support tool for clinical decision.

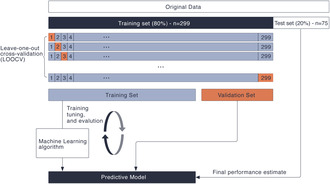

## 1 INTRODUCTION

Orthostatic hypotension (OH) is a prevalent manifestation of autonomic dysfunction in Parkinson's disease (PD), with its incidence varying from 14% to 50% in early‐stage PD patients.^1^, ^2^, ^3^, ^4^ In a recent population‐based study, the odds ratio (OR) of PD was notably high in healthy older subjects (OR = 5.3, 95% CI = 1.4–20.4).^5^ The prominent blood pressure drop resulting from OH can lead to insufficient perfusion in vital organs potentially resulting in issues such as lacunar infarction, cognitive impairment, and gait disorders.^6^, ^7^ Substantial evidence has demonstrated that levodopa and dopamine receptor agonists can trigger or worsen OH in PD patients.^8^, ^9^, ^10^, ^11^, ^12^, ^13^ It was reported that 27% of PD patients had OH during the effectiveness of levodopa/benserazide, while no PD patients had OH in the placebo group.^14^ We hypothesizes that PD patients may be at considerable risk of acute OH following levodopa (AOHPL). However, AOHPL is often asymptomatic and infrequently assessed in routine clinical practice.^3^, ^15^ The hypotensive effect of levodopa ranges from 4.6 to 20 mmHg in systolic blood pressure (SBP) to 2.1–5.0 mmHg in diastolic blood pressure (DBP), predominantly occurring during the effectiveness of levodopa.^10^ OH especially AOHPL stands as a prominent cause of syncope and injurious falls in PD, significantly impairing daily living activities and reducing the overall quality of life.^16^, ^17^ Unrecognized AOHPL may result in inappropriate clinical decisions; therefore, accurate prediction of AOHPL is essential prior to initiating or escalating the dosage of levodopa.

The routine assessment for AOHPL involves the levodopa challenge test (LCT) and the supine‐to‐standing test (STS) during LCT. In our study, all enrolled PD patients underwent LCT and STS hourly during LCT to assess AOHPL using oral levodopa/Benserazide. This evaluation necessitates physicians to monitor blood pressure (BP) via STS and to repeat STS hourly during LCT for a minimum of 3 h. Both LCT and STS are labor‐intensive and time‐consuming, requiring strict adherence to professional operational guidelines, particularly LCT, which mandates the involvement of two well‐trained movement disorder specialists. This complexity makes it challenging for physicians to conduct these tests regularly for every PD patient in their day‐to‐day clinical practice. Consequently, the prediction of AOHPL holds significant importance for physicians as well.

A literature review focusing on OH in PD patients was conducted to pinpoint potential risk factors for consideration in the analyses. Various PD‐related parameters have been reported to elevate the risk of OH in de novo PD patients. These include older age, female gender, lower body mass index (BMI), diabetes mellitus (DM), hypertension, ischemic heart disease, stroke, cognitive impairment, chronic kidney disease, prolonged disease duration, worse disease severity, and akinetic‐rigid type.^5^, ^7^, ^18^, ^19^, ^20^ These potential risk factors are also plausible contributors to AOHPL and align with variables accessible in our dataset (Table S1). In this study, we investigated these factors within an integrated real‐word context. Considering that these factors have been examined independently and separately in prior studies, there may be interactions and significant multicollinearity among them. Hence, a machine learning approach becomes essential to leverage multiple data sources pertaining to fundamental information and clinical disease features encompassing the sequential or temporal trajectory of events recorded in LCT and STS. Thanks to considerable advancements in machine learning, dependable risk predictions in various clinical scenarios have been achieved (i.e., postoperative mortality,^21^ acute kidney injury,^22^ and outcomes of hypertension^23^); therefore, machine learning holds promise in predicting AOHPL, offering potential utility in this context.

The majority of previous studies of OH in PD patients strived to minimize the impact of antiparkinsonian drugs. Few studies have delved into predictive models of OH specifically for PD patients, and as far as our knowledge extends, this study represents the first attempt at implementing a machine‐learning prediction model for predicting AOHPL. The principal objective of this study is to construct and validate an accurate and broadly applicable predictive model for AOHPL. Such a model may empower clinicians to early detection a patient's probability of developing AOHPL and subsequently guide treatment decisions within an outpatient service setting.

## 2 METHODS

### 2.1 Ethics approval and study design

#### 2.1.1 Ethics statement

This study was approved by the Institutional Review Board of Beijing Tiantan Hospital, Capital Medical University and conducted according to the principles of the Declaration of Helsinki. The registration number of this study in Beijing Tiantan Hospital was KYSB2017‐169‐01. All participants provided written informed consent prior to screening and study participation.

#### 2.1.2 Participants

The patient cohort included 497 patients with PD admitted to Beijing Tian Tan Hospital between 2018 and 2019. All subjects were diagnosed as PD by two independent movement‐disorder specialists in accordance with the Movement Disorder Society (MDS) Clinical Diagnostic Criteria.^24^ The inclusion criteria for the AOHPL cohort were (1) OH observed during STS, defined as a sustained orthostatic fall of at least 20 mmHg in SBP or 10 mmHg in DBP within 3 min after rising from a supine to a standing position with or without postural symptoms^6^, ^9^, ^15^; (2) OH occurred during at least one of the three STSs conducted during the LCT after taking levodopa/Benserazide, as outlined in the specific protocol detailed in the Data S1; and (3) patients without severe cognitive impairment who could complete the tests in the study. Patients who did not exhibit OH after levodopa intake were included in the non‐AOHPL group. The exclusion criteria for both groups encompassed (1) use of antihypotensive medications before the trial; (2) allergic to any components of levodopa/Benserazide or mosapride; (3) history of taking anticholinesterase inhibitors, antiarrhythmic drugs, psychotropic agents; and (4) history of renal failure (defined as estimated glomerular filtration rate, eGFR, ≤15).

All the participants underwent a LCT following established guidelines.^25^, ^26^ The LCT was conducted by two movement disorder specialists in the morning, typically between 7 and 8 a.m. Participants were required to fast overnight and abstain from using all dopaminergic medications for at least 12 h prior to the tests. Other medications, including antihypertensive drugs, were maintained during the study antihypertensive medication administered at its usual morning dose. The motor score was assessed through clinical rating based on the Movement Disorder Society‐Unified Parkinson's Disease Rating Scale (MDS‐UPDRS)‐Part III at baseline and at 1‐h intervals following drug administration, for up to 3 h. The STS was performed prior to drug administration and at 1, 2, and 3 h post levodopa/Benserazide ingestion during the LCT. At each of these time points, patients were asked to rest in a supine position for 5 min and then stand up at the bedside for at least 1 and 3 min at most.^27^ Data from each STS were recorded, including supine BP and HR at baseline, as well subsequent standing BP and HR. The largest drop in BP between the supine and standing position was calculated utilizing the lowest recorded standing BP and supine BP in the STS. In cases where patients were unable to tolerate standing, the lowest BP value captured was recorded. BP measurements were taken using an automated sphygmomanometer (Omron HEM‐7312, Omron Healthcare Co. Ltd.) positioned at heart level on the left arm. Each BP reading was taken twice, and the readings were averaged to determine supine BP values. Additional methodological details can be found in the Data S1.

### 2.2 Statistical analysis

Complete case was applied to address missing data. Statistical significance level was established at 0.05. Statistical analysis was conducted by R‐4.0.2 software.

The *Shapiro–Wilk test* was utilized to assess whether continuous variables adhered to a normal distribution. Normally distributed variables were presented as the mean (standard deviation) and compared between the AOHPL and non‐AOHPL groups using unpaired two‐tailed *student's t tests*. Non‐normally distributed variables were depicted as the median (the interquartile range (IQR)), and disparities between the AOHPL and non‐AOHPL groups were evaluated utilizing the *Wilcoxon rank sum test*. Categorical variables were presented as numbers (percentages), and comparisons between the two groups were conducted using the *chi‐square test*.

After identifying variables that exhibited significant differences between groups, *binary logistic regression* was conducted using each of these variable as independent variables, with the absence or presence of AOHPL serving as the dependent variable. The discriminatory ability of each variable was evaluated using the area under the receiver operating characteristic curve (AUC_ROC), where a higher AUC_ROC value signifies a better discriminative ability.

Univariate odds ratios (ORs) along with their corresponding 95% confidence intervals (CIs) were utilized to quantify the risk of AOHPL associated with each individual variable. For continuous variables, the ORs represent the change in odds of the presence of AOHPL for each unit increase in the variable. For categorical variables, the ORs illustrate the change in odds of the presence of AOHPL in comparison to the chosen reference group.

AUC_ROC, accuracy, Cohen's Kappa, sensitivity, specificity, positive predictive value (PPV), and negative predictive value (NPV) were used to evaluate machine learning model performance in both training set and independent test set. We set AOHPL as a positive case and non‐AOHPL as a negative case. Accuracy was the ability to classify patients as AOHPL and non‐AOHPL correctly; Cohen's Kappa coefficient was calculated to assess the agreement between the model prediction and the true cases. Sensitivity (recall) was its ability to classify AOHPL correctly, while specificity was its ability to classify non‐AOHPL correctly. PPV (precision) was the probability that a participant was predicted as AOHPL actually was AOHPL, while NPV was the probability that a participant predicted as non‐AOHPL truly was not non‐AOHPL. AUC_ROC (from 0 to 1) was a metric that could be used to qualify the overall discriminative ability of our model and summarized the trade‐off between the sensitivity and false positive rate (1 − specificity) for a predictive model using different probability thresholds, and it was appropriate when the observations were balanced between each group.

### 2.3 Model construction and evaluation

#### 2.3.1 Training and independent test data split

The whole dataset underwent an initial exclusion of 113 patients with missing data, stratified by AOHPL or non‐AOHPL. Subsequently, the dataset was randomly divided into a training set and a test set, allocating 80% of the entire data for training, reserving 20% of the entire data for independent test data evaluation. Feature analysis and selection were conducted using the training data. Leave‐one‐out cross‐validation (LOOCV) was performed as a validation method in the training set to tune the hyperparameters and estimate the performance of the constructed predictive model. Independent test data were then utilized to furnish an unbiased evaluation of the final model that was established using the training set (Figure S1).

#### 2.3.2 Feature selection

Due to the interdependent nature of (1) standing SBP, standing DBP, and standing MAP; (2) supine SBP, supine DBP, and supine MAP; (3) ΔSBP, ΔDBP, and ΔMAP; (4) MMSE and MOCA; (5) LEU of overall antiparkinsonian drugs; and (6) LEU of Madopar and Sinemet and the dose of levodopa used in an LCT, only standing MAP, Supine MAP, ΔSBP, ΔDBP, MOCA and the dose of levodopa used in an LCT were kept. In addition, predrug OH is defined based on ΔSBP and ΔDBP, and due to the interdependent nature, the inclusion of ΔSBP, ΔDBP can represent the predrug OH status of the subject. These six features, alongside other demographic, clinical symptoms, and drug information (for a total of 21 features), were transferred to the next step, feature selection. Utilizing the random forest technique, feature permutation importance was assessed to select the most influential features from this pool of 21. The first K features showing the largest mean decrease Gini were chosen for subsequent predictive model construction.

#### 2.3.3 Predictive model for discrimination between AOHPL and non‐AOHPL

After completing feature selection, random forest, support vector machine (SVM), and logistic regression techniques were employed to build classification models for predicting whether a patient should be classified as AOHPL or non‐AOHPL. The performance of predictive models with features from 1 to 21 (in steps of 1) for K was examined and compared to ensure that we could obtain the best predictive model. The performance of these predictive models was evaluated using several metrics including AUC_ROC, accuracy, Kappa, sensitivity, specificity, PPV, and NPV. By comparing the model performance across different feature sets ranging from 1 to 21, the objective was to identify the combination of features that would yield the most effective predictive model.

#### 2.3.4 Independent test data evaluation

Independent test data were used as a validation cohort to evaluate the final predictive model performance and ensure its generalization ability. The model's performance was gauged using a range of evaluation metrics including AUC_ROC, accuracy, Kappa, sensitivity, specificity, PPV, and NPV.

Local interpretation was performed on a given observation and associated prediction to determine how variables explain this specific prediction using Shapley additive explanations (SHAP) values.^28^ The predicted probability of each observation obtained from the final predictive model was stratified into two components, the baseline value and the feature contribution of each feature included in the model construction. The baseline value referred to the average predicted probability of AOHPL in the training dataset.

#### 2.3.5 Additional validation

The additional validation involving 10 rounds of random splitting for training and testing sets was performed to assess the model's robustness. The purpose was to evaluate the overall consistency of the model's performance across multiple iterations and to check its reliability under varying data subsets.

## 3 RESULTS

### 3.1 Participant characteristics

A total of 497 patients participated in this study. The demographic and clinical characteristics of the participants are presented in Table S1. The number of participants with missing data for potential predictors or outcomes was 113. Complete case analysis was applied to address the missing data. After excluding these 113 patients in our analysis, we had 374 participants in total included in our analysis. The main demographic characteristics of the 374 participants included in our analysis are shown in Table 1. AOHPL and non‐AOHPL participants were significantly different in age, LEU of Madopar and Sinemet, presence of supine hypertension or not, presence of dizziness or not, presence of predrug OH or not, ΔSBP, ΔDBP, ΔMAP, and max ΔSBP Post‐Drug, and max ΔDBP Post‐Drug.

**TABLE 1:** Main characteristics of the participants.

	All	NON‐AOHPL	AOHPL	*p*
*N*	374	180	194	
Age, years	65.00 [58.00, 70.00]	62.50 [56.00, 69.00]	66.00 [61.00, 70.00]	**0.002**
Sex: Male, *n* (%)	224 (59.9)	107 (59.4)	117 (60.3)	0.948
BMI, kg/m^2^	24.16 (3.55)	24.27 (3.83)	24.07 (3.28)	0.587
Use antihypertensive drugs, *n* (%)	101 (27.0)	46 (25.6)	55 (28.4)	0.623
DM, *n* (%)	53 (14.2)	19 (10.6)	34 (17.5)	**0.075**
Disease course, years	6.00 [3.00, 9.00]	6.00 [3.00, 8.00]	6.00 [4.00, 10.00]	0.288
MDS‐UPDRS III (OFF)	38.00 [27.00, 52.00]	37.00 [26.00, 50.00]	39.50 [29.00, 54.00]	0.103
Subtype (%)				
TD	219 (58.6)	103 (57.2)	116 (59.8)	0.575
PIGD	131 (35.0)	63 (35.0)	68 (35.1)	
Indeterminate	24 (6.4)	14 (7.8)	10 (5.2)	
pre‐drug OH	122 (32.6)	27 (15.0)	95 (49.0)	**<0.001**
Supine hypertension, *n* (%)	197 (52.7)	76 (42.2)	121 (62.4)	**<0.001**
Dyskinesia, *n* (%)	80 (21.4)	34 (18.9)	46 (23.7)	0.312
Dizziness, *n* (%)	40 (10.7)	11 (6.1)	29 (14.9)	**0.009**
MMSE	27.00 [24.00, 28.00]	27.00 [24.00, 29.00]	26.00 [23.00, 28.00]	0.130
MoCA	21.50 [17.00, 25.00]	22.00 [18.00, 25.00]	21.00 [17.00, 24.00]	0.097
Drugs				
LEU of overall antiparkinsonian drugs	500.00 [228.12, 750.00]	450.00 [187.50, 700.00]	537.50 [253.12, 775.00]	0.158
LEU of Madopar and Sinemet	280.00 [120.00, 454.69]	240.00 [118.12, 400.00]	296.25 [160.00, 480.00]	**0.037**
Dopamine receptor agonists, mg	37.50 [0.00, 75.00]	37.50 [0.00, 75.00]	37.50 [0.00, 100.00]	0.532
MAO‐B, mg	0.00 [0.00, 0.00]	0.00 [0.00, 0.00]	0.00 [0.00, 0.00]	0.972
Benzhexol, mg	0.00 [0.00, 0.00]	0.00 [0.00, 0.00]	0.00 [0.00, 0.00]	0.711
Amantadine	0.00 [0.00, 100.00]	0.00 [0.00, 100.00]	0.00 [0.00, 100.00]	0.192
The dose of levodopa used in an LCT, mg	125.00 [62.50, 187.50]	125.00 [62.50, 187.50]	125.00 [62.50, 187.50]	0.158
Hemodynamics				
Supine SBP, mmHg	136.61 (18.77)	132.51 (17.77)	140.43 (18.91)	**<0.001**
Standing SBP, mmHg	124.00 [114.00, 138.00]	128.00 [115.00, 141.00]	121.00 [109.00, 134.75]	**0.001**
Supine DBP, mmHg	82.00 [77.00, 89.75]	80.00 [75.00, 87.00]	84.00 [78.00, 90.75]	**<0.001**
Standing DBP, mmHg	81.00 [74.00, 88.75]	83.00 [77.00, 90.00]	79.00 [73.00, 88.00]	**0.001**
ΔSBP, mmHg	9.00 [−0.75, 22.00]	2.00 [−4.00, 10.00]	17.50 [6.25, 27.00]	**<0.001**
ΔDBP, mmHg	0.00 [−4.00, 7.00]	−3.00 [−7.25, 2.25]	4.00 [−1.75, 10.75]	**<0.001**
Supine MAP, mmHg	101.00 [93.33, 108.25]	98.00 [91.25, 105.75]	103.00 [95.67, 110.25]	**<0.001**
Standing MAP, mmHg	96.67 [87.33, 103.33]	98.17 [90.08, 105.75]	93.67 [86.08, 101.33]	**<0.001**
ΔMAP, mmHg	3.67 [−3.00, 10.00]	−0.67 [−5.08, 5.00]	8.50 [2.00, 15.67]	**<0.001**
Supine HR	74.22 (12.10)	72.44 (11.94)	77.20 (12.19)	0.233
Standing HR	81.40 (13.14)	78.64 (13.24)	86.00 (11.99)	0.086
ΔHR	7.18 (8.04)	6.20 (8.85)	8.80 (6.44)	0.329
max ΔSBP Post Drug, mmHg	16.00 [7.00, 29.00]	8.00 [3.00, 13.00]	28.00 [21.00, 37.00]	**<0.001**
max ΔDBP Post Drug, mmHg	6.00 [1.00, 13.00]	2.00 [−2.00, 4.25]	13.00 [7.00, 19.00]	**<0.001**
Post‐drug ΔSBP compared with that of pre‐drug, *n* (%)				
Better	34 (9.1)	22 (12.2)	12 (6.2)	**<0.001**
Similar	180 (48.3)	103 (57.2)	77 (39.9)	
Worse	159 (42.6)	55 (30.6)	104 (53.9)	
Post‐drug ΔDBP compared with that of pre‐drug, *n* (%)				
Better	44 (11.8)	26 (14.4)	18 (9.3)	**<0.001**
Similar	122 (32.6)	74 (41.1)	48 (24.7)	
Worse	208 (55.6)	80 (44.4)	128 (66.0)	

*Note*: Data are shown as the mean (SD) for normally distributed continuous variables, median (IQR) for nonnormally distributed continuous variables, and *n* (%) for categorical variables. Supine hypertension: a SBP of ≥140 and/or DBP of ≥90 mmHg. max ΔSBP Post‐drug: Maximum change in systolic blood pressure within 3 h after taking medication. max ΔDBP Post‐drug: Maximum change in diastolic blood pressure within 3 h after taking medication. Post‐drug ΔSBP and ΔDBP compared with that of pre‐drug: a worse status with difference between max ΔSBP Post‐drug and ΔSBP being ≥10 mmHg, a similar status with difference between max ΔSBP Post‐drug and |ΔSBP| < 10 mmHg, a better status with difference between max ΔSBP Post‐drug and ΔSBP≤‐10 mmHg. Similarly, the threshold for ΔDBP was 5 mmHg.

*p* values less than 0.05 were bolded, indicating significant differences between the two groups.

*Abbreviations*: AOHPL, post antiparkinsonian drug OH; BMI, body mass index, DM, diabetes mellitus; HR, hear rate; LCT, acute levodopa challenge tests; LEU, daily levodopa equivalent unit; MAO‐B, monoamine oxidase‐B; MDS‐UPDRS‐III, Movement Disorders Society‐Unified Parkinson's Disease Rating Scale part 3; MMSE, mini‐mental state examination; MoCA, Montreal Cognitive Assessment; PIGD, postural instability and gait disorders; TD, tremor dominant.

Table S2 and Figure S2 display the changing trends in hemodynamic indices. After taking levodopa, 22% (27/122) of patients who initially experienced predrug OH did not encounter AOHPL, while 39% (99/252) of patients without predrug OH experienced AOHPL. The occurrence of pre‐drug OH and AOHPL are not independent (χ^2^ = 1289.3, *p* < 0.05, Table S3). In the AOHPL group, 51% (99/194) patients did not have predrug OH. There were 15% (27/180) patients had OH at baseline in the non‐AOHPL group. Moreover, the post‐drug changes in ΔSBP and ΔDBP were more pronounced in the AOHPL group.

There was not a widely accepted definition about degree of OH after levodopa compared to their baseline (predrug) OH. In this study, we defined 3 status of post‐drug ΔSBP and ΔDBP compared with that of predrug. A worse status was difference between max ΔSBP post‐drug and ΔSBP being ≥10 mmHg. A similar status was difference between max ΔSBP Post‐drug and |ΔSBP| < 10 mmHg. And a better status was difference between max ΔSBP post‐drug and ΔSBP ≤ −10 mmHg. Similarly, the threshold for ΔDBP was 5 mmHg. Specifically, 53.9% (104/194) and 66.0% (128/194) patients within this group experienced worsening post‐drug ΔDBP and post‐drug ΔSBP, respectively. The 95% CI for the difference between post‐drug ΔSBP and predrug ΔSBP was [9.53, 13.944], and for the difference between post‐drug ΔDBP and predrug ΔDBP, it was [7.508, 11.069].

The comparison of independent test data and the training data of the distribution of the main demographic features are shown in Tables S4 and S5 for non‐AOHPL and AOHPL respectively. There was no statistically significant difference in most main demographic features between the training data and test data.

### 3.2 Feature analysis results

In bivariate analysis, 13 features were significantly different between the non‐AOHPL and AOHPL groups (Table 2). For example, participants with AOHPL were 5.6% older than non‐AOHPL on average. The ΔMAP of participants with AOHPL was 7.3 mmHg higher than that of participants with non‐AOHPL on average. Feature discriminative ability in differentiating AOHPL and non‐AOHPL was evaluated by individual AUCs. Three out of 13 statistically significant features had AUCs larger than 0.7. Table 2 also shows the results of univariate analysis. For example, regarding age (Univariate OR = 1.041, 95% CI = 1.016–1.067), the increase in odds of AOHPL occurrence was 4.1% for each unit (year) increase in age without considering other factors. Regarding dizziness (Univariate OR = 2.335, 95% CI = 1.070–5.095), the odds of AOHPL occurring in participants with dizziness was 2.34 times that in participants without dizziness while not considering other factors.

**TABLE 2:** Comparison of features between AOHPL and non‐AOHPL on training data.

	NON‐AOHPL	AOHPL	*p*	Auc	Univariate OR (95% CI)
*N*	144	155			
Age, years	62.50 [56.00, 69.00]	66.00 [61.00, 71.00]	0.003	0.599	1.041 (1.016–1.067)
Sex: Male, *n* (%)	88 (61.1)	95 (61.3)	1.000		
BMI, kg/m^2^	24.22 (3.83)	23.97 (3.32)	0.544		
Use antihypertensive drugs, *n* (%)	34 (23.6)	44 (28.4)	0.419		
DM, *n* (%)	13 (9.0)	28 (18.1)	0.036	0.545	2.222 (1.101–4.481)
Disease course, years	6.00 [3.00, 8.00]	6.00 [4.00, 9.50]	0.474		
MDS‐UPDRS III (OFF)	37.00 [26.00, 46.25]	39.00 [28.50, 54.00]	0.121		
pre‐drug OH	22 (15.3)	75 (48.4)	0.001	0.666	5.199 (2.992–9.034)
Subtype (%)					
TD	80 (55.6)	95 (61.3)	0.256		
PIGD	51 (35.4)	53 (34.2)			
Indeterminate	13 (9.0)	7 (4.5)			
Dyskinesia, *n* (%)	31 (21.5)	41 (26.5)	0.390		
Dizziness, *n* (%)	10 (6.9)	23 (14.8)	0.046	0.539	2.335 (1.07–5.095)
Supine hypertension, *n* (%)	58 (40.3)	98 (63.2)	0.001	0.615	2.549 (1.599–4.064)
MMSE	27.00 [24.00, 29.00]	27.00 [23.00, 28.50]	0.251		
MoCA	22.00 [18.00, 25.00]	21.00 [17.50, 24.00]	0.241		
Drugs					
LEU of overall antiparkinsonian drugs	475.00 [268.75, 703.12]	540.50 [268.75, 756.25]	0.333		
LEU of Madopar and Sinemet	288.12 [120.00, 410.62]	320.00 [160.00, 480.00]	0.176		
Dopamine receptor agonists, mg	37.50 [0.00, 75.00]	37.50 [0.00, 100.00]	0.406		
MAO‐B, mg	0.00 [0.00, 0.00]	0.00 [0.00, 0.00]	0.846		
Benzhexol, mg	0.00 [0.00, 0.00]	0.00 [0.00, 0.00]	0.492		
Amantadine	0.00 [0.00, 100.00]	0.00 [0.00, 100.00]	0.368		
The dose of levodopa used in an LCT, mg	125.00 [62.50, 187.50]	125.00 [62.50, 187.50]	0.506		
Hemodynamics					
Supine SBP, mmHg	131.93 (17.67)	140.14 (19.40)	0.001	0.623	1.024 (1.011–1.038)
Standing SBP, mmHg	127.50 [115.75, 139.25]	121.00 [108.00, 133.00]	0.002	0.603	0.983 (0.971–0.995)
Supine DBP, mmHg	80.00 [74.75, 87.00]	84.00 [78.00, 90.00]	0.004	0.597	1.036 (1.014–1.059)
Standing DBP, mmHg	82.50 [77.00, 89.00]	79.00 [73.00, 88.00]	0.015	0.582	0.979 (0.959–1)
ΔSBP, mmHg	3.00 [−2.00, 10.00]	18.00 [5.50, 26.00]	0.001	0.767	1.084 (1.06–1.108)
ΔDBP, mmHg	−3.00 [−6.25, 3.00]	3.00 [−1.50, 9.50]	0.001	0.701	1.074 (1.044–1.104)
Supine MAP, mmHg	98.00 [91.00, 104.83]	102.33 [95.67, 109.17]	0.001	0.619	1.038 (1.017–1.058)
Standing MAP, mmHg	97.67 [89.17, 104.42]	93.00 [85.50, 101.33]	0.003	0.601	0.975 (0.957–0.994)
ΔMAP, mmHg	0.00 [−4.17, 5.08]	7.33 [2.17, 14.83]	0.001	0.757	1.109 (1.075–1.144)

*Note*: Data are shown as the mean (SD) for normally distributed continuous variables, median [Lower Quartile Part (Q1), Upper Quartile Part (Q3)] for nonnormally distributed continuous variables, and *n* (%) for categorical variables.

*Abbreviations*: AOHPL, post antiparkinsonian drug OH; BMI, body mass index; DBP, diastolic blood pressure; DM, diabetes mellitus; HT, hypertension; LCT, acute levodopa challenge tests; LEU, daily levodopa equivalent unit; MAO‐B, monoamine oxidase‐B; MDS‐UPDRS‐III, Movement Disorders Society‐Unified Parkinson's Disease Rating Scale part 3; MMSE, mini‐mental state examination; MoCA, Montreal Cognitive Assessment; PIGD, postural instability and gait disorders; SBP, systolic blood pressure; TD, tremor dominant.

### 3.3 Model performance and interpretation

The discrimination performance of the predictive model comparisons based on the LOOCV approach on the training data is shown in Table 3. It displays the best model among logistic regression, SVM, and random forest. The random forest algorithm‐based model outperformed the other two models. Table 4 presents the mean decrease Gini (MD Gini) of the features in the final random forest predictive model construction. The total number of features included in the model is 17. The results show that ΔSBP has the highest MD Gini, which means that it is the most important feature for constructing the predictive model. The dose of levodopa used in an LCT was not significantly different between the non‐AOHPL and AOHPL groups (Table 2, *p* = 0.506); however, it contributed to model construction when combined with other factors. The dose of levodopa was the identification parameter in LCT. A higher dosage of levodopa was associated with more severe hypotension effects and a higher incidence of OH.^29^, ^30^

**TABLE 3:** Model comparisons.

Type	Fea_num	AUC_ROC	Accuracy	Kappa	Sensitivity	Specificity	PPV	NPV
LR	13	0.747	68.6%	0.371	69.0%	68.1%	69.9%	67.1%
SVM	18	0.759	71.2%	0.425	69.7%	72.9%	73.5%	69.1%
RF	17	0.776	73.6%	0.472	71.6%	75.7%	76.0%	71.2%

*Abbreviations*: AUC_ROC, the area under the receiver operating characteristic curve; Fea_num, the number of features included in model construction; LR, logistic regression; NPV, negative predictive value; PPV, positive predictive value; RF, random forest; SVM, support vector machine.

**TABLE 4:** Mean decrease in the Gini and mean of absolute SHAP value of features for model construction.

	MeanDecreaseGini	Mean of absolute SHAP value
ΔSBP	26.11103732	0.133454667
ΔDBP	16.04391543	0.081726667
Supine MAP	13.52732932	0.039872
Standing MAP	13.17802189	0.035852
BMI	11.38210534	0.008186667
Age	11.27249855	0.035630667
MDS‐UPDRS III (OFF)	10.34680449	0.009522667
MoCA	9.068037098	0.01058
Disease course	8.189009409	0.008102667
Dopamine receptor agonists	6.245385682	0.009050667
The dose of levodopa used in an LCT	3.758917767	0.004368
Subtype	3.345599979	0.003510667
Supine hypertension	2.958345969	0.036596
Amantadine	2.890750141	0.006108
Benzhexol	2.155844992	0.003781333
DM	1.7342645	0.009013333
Sex	1.533873644	0.00072

*Note*: Mean of Absolute SHAP Value: calculated in all test cases.

*Abbreviations*: BMI, body mass index; DBP, diastolic blood pressure; DM, diabetes mellitus; LCT, acute levodopa challenge test; MoCA, Montreal Cognitive Assessment; SBP, systolic blood pressure.

**FIGURE 1:**
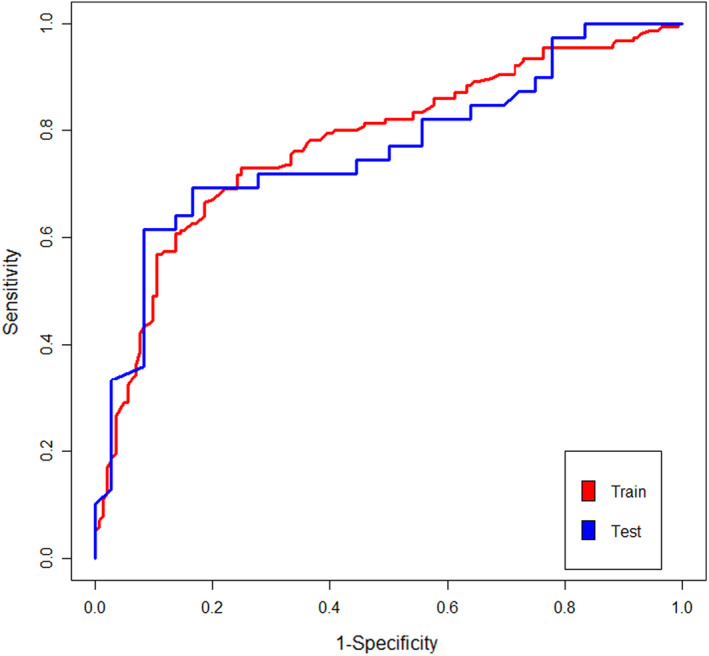
Receiver operating characteristic curves (ROCs) of the predictive model. The red line depicts model performance based on the leave‐one‐out cross‐validation (LOOCV) approach on the training data, while the blue line depicts model performance on independent test data after hyperparameter tuning and training.

The final predictive random forest model was evaluated on the independent test data. Model performance on the independent test data (accuracy 72%, Kappa 0.440, sensitivity 71.8%, specificity 72.2%, PPV 73.7%, NPV 70.3%, and AUC_ROC 0.764) was nearly as good as the LOOCV result with the training data (accuracy 73.6%, Kappa 0.472, sensitivity 71.6%, specificity 75.7%, PPV 76%, NPV 71.2%, and AUC_ROC 0.776; Figures 1 and S3). It represents the generalization ability of our predictive model. Figure S3 shows the confusion matrix tables of the random forest model on the training data and test data. In our study, the discriminative ability of a predictive model referred to the ability to correctly classify participants as AOHPL or non‐AOHPL. AUC_ROC was a metric that could be used to qualify the overall discriminative ability of our model over a range of all possible classification thresholds (from 0 to 1; Figure 1).

To further validate the model's robustness, we conducted an additional 10 rounds of random splitting for training and testing sets and evaluated the overall performance of the model (Table S6). The results from the training set across the 10 random splits were consistent with the previous report. However, for the independent testing set, the corresponding performance varied from that observed on the initial independent test data (accuracy 61%, Kappa 0.21, sensitivity 81%, specificity 31%, PPV 58%, NPV 70%, and AUC_ROC 0.72). This discrepancy suggests the need for further investigation into the model's generalizability and potential variations in predictive performance across different test sets.

Local interpretation was performed on all individual observations on independent test data (75 cases in total) with SHAP values to disclose how variables explain the predictive AOHPL probability. Table S7 shows that for specific observations, we identify the three variables with the most impact on the prediction of AOHPL. For example, for patient ID 70, ΔSBP (value: 8, impact: −0.118), age (value: 59, impact: −0.058), and without supine hypertension (impact: −0.053) decreased the probability of AOHPL. Table S8 shows the ranking of variables according to which are the most frequent in the top three so that we can know which variables are most explanatory in the prediction. ΔSBP was the most contributing variable for the prediction in 50 cases and the second most contributing variable in 23 cases and the third in 2 cases. ΔDBP was the most contributing variable for the prediction in 24 cases, the second in 27 cases, and the third in 10 cases. Standing MAP was the second most contributing variable for the prediction in 9 cases and the third in 17 cases.

**FIGURE 2:**
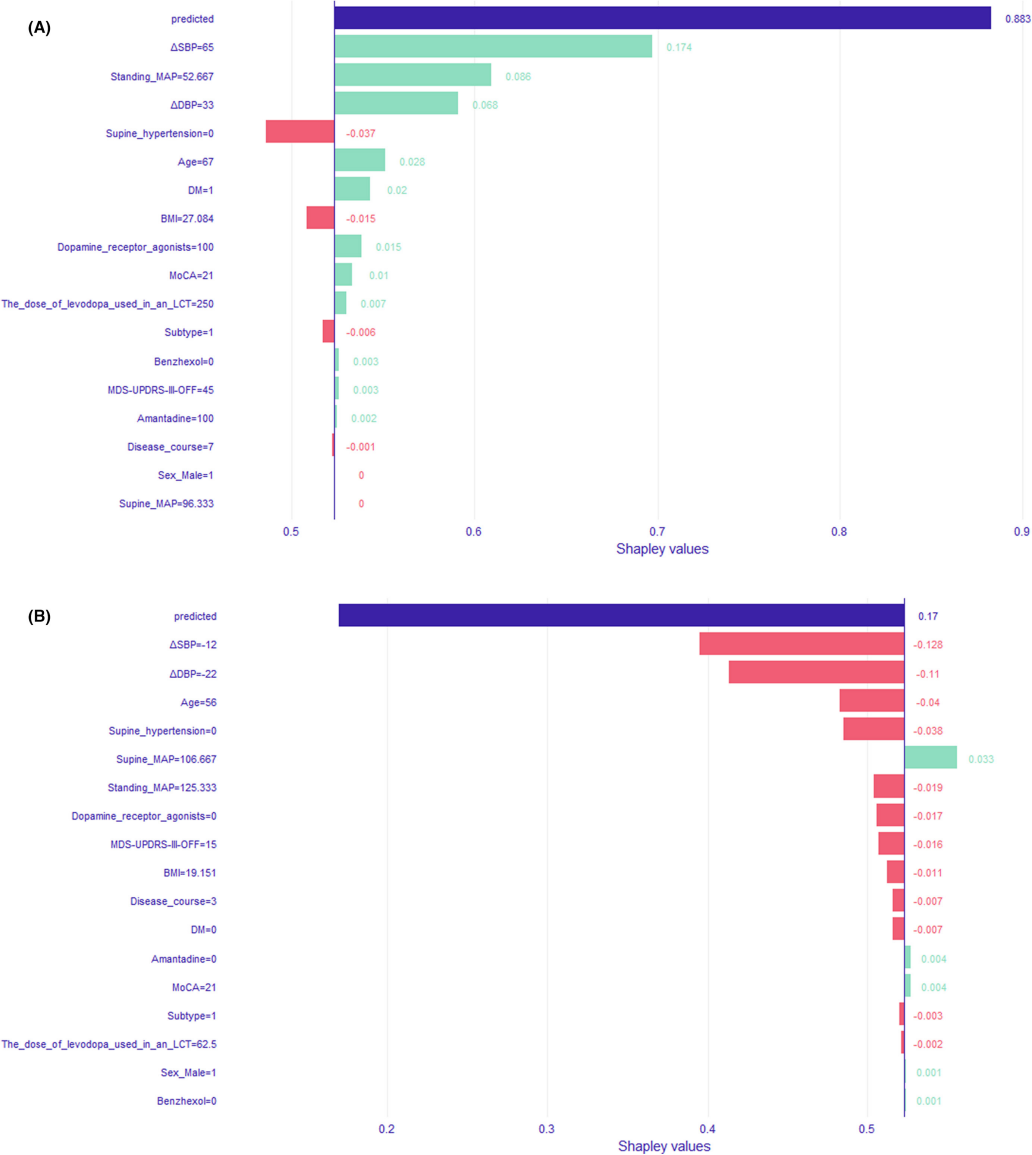
SHAP plot of two specific patients with ID 54 (AOHPL case) and ID 63(non‐AOHPL case). (A) is the SHAP plot for patient with ID 54 (AOHPL case) with probability of AOHPL (0.883) = baseline prob (0.523) + the feature contribution of each feature included in the model construction (0.174 + 0.086 + 0.068–0.037 + 0.028 + 0.021–0.015 + 0.015 + 0.01 + 0.007–0.006 + 0.003 + 0.003 + 0.002); (B) is the SHAP plot for patient with ID 63(non‐AOHPL case) with probability of AOHPL (0.17) = baseline prob (0.523) + the feature contribution of each feature included in the model construction (−0.128–0.11 – 0.04 – 0.038 + 0.033 – 0.019 – 0.017 – 0.016 – 0.011 – 0.007 + 0.004 + 0.004 – 0.003 – 0.002 + 0.001 + 0.001).

Figure 2 shows the SHAP plots of patient with ID 54 (AOHPL Case, Figure 2A) and patient with ID 63 (non‐AOHPL Case, Figure 2B), respectively. The label on the y‐axis shows the values of the model‐constructed predictors for this particular patient. The purple bar shows the predicted probability of AOHPL for this particular patient, and the green and red bars show the effect of each variable on this prediction (red: decreased probability, green: increased probability). The effects of all variables and the baseline value 0.523 sum up to the value of the predicted probability of AOHPL for this particular patient. We can see that the dose of levodopa used in an LCT (250 mg) increased the probability of AOHPL for patient with ID 54 (prob: +0.007), while it (62.5 mg) decreased the probability of AOHPL for patient with ID 63 (prob: −0.002). Age (56 years) decreased the probability of AOHPL for patient with ID 63 (prob: −0.04), while age (67 years) increased the probability of AOHPL for patient with ID 54 (prob: +0.028).

## 4 DISCUSSION

In our study, we developed a random forest predictive model to predict whether a PD patient would have AOHPL following the introduction of levodopa treatment or not. To the best of our knowledge, this is the first study to use a machine‐learning algorithm to create a model for prediction of AOHPL in PD patients. This accurate and generalizable predictive model uses 17 widely available parameters to handle high‐dimensional data before levodopa administration. It was evaluated with independent test data to present its generalization. This approach would avoid screening tests for AOHPL, with accuracy of 73.6%, sensitivity of 71.6%, and specificity of 75.7%. Physicians could put proposed levodopa therapeutic regimen, individual information of PD patients into the prediction model and get the probability of AOHPL, along with impact of each feature. It can individually instruct physicians and PD patients to avoid AOHPL from results given by the model (i.e., Figure 2). We proposed that a dosage titration therapy of levodopa in the PD patients whose probability of AOHPL were positive (*p* > 0.5) in screening results according to their clinical information before levodopa administration. The clinical utility of this approach is threefold: (1) to improve the early recognition of AOHPL, the result of AOHPL predictive model can provide PD patients with an adequate therapeutic regimen of levodopa. If the patient has a high probability of AOHPL and levodopa is essential, different dosages of levodopa and combination of antiparkinsonian drugs can be put into the predictive model and find a least harmful treatment option for the patient, and assessments of AOHPL are suggested before medication; (2) to enhance prospective AOHPL estimation, by the use of this model physicians can assess the probability of having AOHPL case‐by‐case and tailor the diagnostic workup for each individual PD patient, alleviating clinical workload for physicians; and (3) to provide mechanistic insights into the hypotensive effects of levodopa in PD.

The most prominent parameters of our AOHPL prediction model were ΔSBP, ΔDBP, supine MAP, standing MAP, BMI, and age. They were the most important features for discriminating AOHPL from non‐ AOHPL based on the mean decreased Gini metric (Table 4). We also used SHAP values to determine how variables explain this specific prediction for each subject in the independent test data. For most of the subjects, ΔSBP, ΔDBP, and standing MAP ranked as the top 3 strongest predictors (Table S8).

ΔSBP and ΔDBP before medication were the most contribution features for the model construction (Table 4). Consistent with previous studies, OH in PD patients was part of the disease process.^13^, ^31^ Mechanisms of OH in PD included involvement of both central and peripheral components of the autonomic nervous system, such as preganglionic sympathetic neurons and paravertebral autonomic ganglia.^32^, ^33^ In addition to an inherent component of autonomic dysfunction in PD, AOHPL also can be an adverse effect of Levodopa/benserazide which is widely used in PD patients.^12^, ^30^, ^32^ As our model showed that it was complicated, multiple factors were integrated. The probability of AOHPL was individually analyzed in patients, for ID 61 ΔSBP, supine hypertension and standing MAP contributed to the probability of AOHPL most; while for ID 70 ΔSBP, age and supine hypertension were the first three variables contributed most (Table S7). Benserazide is a peripheral decarboxylase inhibitor (DCI), which has no evidence to effect blood pressure so far.^32^, ^34^ Substantial data showed that levodopa regulated hemodynamic change via binding to and activating dopamine receptors in both peripheral and central mechanisms.^12^, ^13^, ^32^, ^35^ In the setting of decreased cardiovascular sympathetic innervation and baroreflex failure, levodopa induced cardioinhibitory and hampered the efficiency of reflex vasoconstriction in patients with PD.^13^, ^14^, ^30^, ^36^ For example, in one study, LCT of 200 mg levodopa/50 mg Benserazide evoked a significant decrease in MAP (−15%, *p* < 0.001), cardiac stroke volume (−13%, *p* < 0.01), and inotropism (dP/dt: −18%, *p* < 0.001).^36^ At a central level, it decreased the activity of vasopressor areas within the brainstem and markedly denervation of catecholaminergic neurons in the site of the initial synapse of baroreflexes.^30^, ^37^, ^38^, ^39^, ^40^

In this study, supine/standing MAP and supine hypertension were important features for discriminating AOHPL from non‐AOHPL based on the mean decreased Gini metric (Table 4). The level of MAP represents the perfusion of vital organs. Maintenance of MAP below 75 mmHg would be insufficient to perfuse cerebral tissues. Previous studies used standing MAP ≤ 75 mmHg as an indicator of clinically significant OH.^9^, ^29^ And they found that standing MAP provided a very high sensitivity (97.2%) and specificity (98.3%) in discriminating symptomatic OH (i.e., dizziness and fainting).^9^ Complementary to previous studies, supine MAP and supine hypertension were also crucial indicators for AOHPL in this study. Supine MAP (Univariate OR = 1.038, 95% CI = 1.017–1.058; see Table 2) indicated that the increasing in odds of AOHPL occurrence was 1.7% for each unit (mmHg) increased in supine MAP before drug without considering other factors. The odds of AOHPL occurring in participants with supine hypertension were 2.55 times higher than in participants without supine hypertension (Univariate OR = 2.549, 95% CI = 1.599–4.064). Supine hypertension and OH commonly coexist in PD patients, and more than 50% of OH patients have supine hypertension.^6^, ^41^, ^42^ This dilemma may be caused by cardiac and extracardiac sympathetic denervation or baroreflex failure.^37^, ^43^ The paradox hemodynamics may misguide physicians to allow management of supine hypertension based on merely sitting or supine BP measurements. Improvement of supine hypertension may be at the expense of worsening OH, which might be associated with more urgent complications.

Age is a generally acknowledged risk factor for OH in normal subjects and PD patients.^9^ Elderly PD patients are at higher risk of OH and may suffer worse orthostatic BP decreases during levodopa therapy. In this study, when we considered age individually, for PD patients, 1 year older could increase the odds of AOHPL occurrence by 4.1% (univariate OR = 1.041, 95% CI = 1.016–1.067). The “triple whammy” hypothesis suggested three possible pathophysiological causes related to catecholaminergic deficiency in PD patients with OH, including noradrenergic denervation, the vestibulosympathetic reflex, and arterial baroreflex failure.^6^, ^8^, ^43^ All three of these causes are associated with aging.^41^

Several machine‐learning algorithms were tried in our study to achieve the goal of constructing a predictive classification model to distinguish between AOHPL and non‐AOHPL. The algorithms included logistic regression, SVM, and random forest. Logistic regression is easier to implement and interpret; however, it has linear boundaries. SVM usually performs well in high‐dimensional space; however, we could not explain the classification in terms of probability as the SVM‐based model was not a probabilistic model. Random forest can reduce the overfitting problem of the decision tree method and is stable; however, it is complex and time‐consuming to train. All three algorithms have their advantages and drawbacks. Therefore, the models were tested, and model performance was compared in our study. Random forest was chosen as our final modeling approach because it outperformed the other two methods.

The random forest technique is highly flexible and considers interaction effects between variables with relatively low bias. Random forest is an ensemble learning method that builds hundreds of trees with randomly selected records and variables and takes the majority vote from these trees. Our predictive model was built with hyperparameter tuning on training data through the LOOCV approach and then validated with independent test data. Our random forest model was built with the goal of increasing the predictive accuracy at the cost of reducing the interpretability of the model and variables for constructing the model. The hyperparameters tuned with the grid search method were considered among the following algorithm parameters: the number of variables to be randomly sampled as candidates at each node split for a tree, the maximum number of terminal nodes allowed in a tree, the number of trees, and the minimum number of terminal nodes that should be held in a tree. In our study, a predictive case was set as AOHPL when the predictive probability exceeded the preset threshold; otherwise, it was set as non‐AOHPL. We obtained our model performance based on the classification threshold of 0.5, which is a common default threshold for classification models. Actually, the threshold value could be chosen by clinicians depending on their prioritization of either sensitivity or specificity, which corresponds to their preference regarding prioritization of either the ability to classify a subject with AOHPL correctly or the ability to classify a subject with non‐AOHPL correctly.

### 4.1 Limitation

Some limitations and future work need to be considered in order to improve the current study. There are individual differences in clinical features, even among participants who are in the same group (AOHPL or non‐AOHPL). Future research should include a larger number of participants in order to better represent the population and increase the reliability and accuracy of the research. In addition to the SVM, random forest, and logistic regression models, other models should also be considered to potentially improve the accuracy of the current classification framework.

## AUTHOR CONTRIBUTIONS

Manuscript preparation, writing of the first draft and research project: Zhu Liu, Shinuan Lin, Tao Feng, and Yun Ling. Data preprocessing and statistical analysis: Shinuan Lin. Execution: Xuemei Wang, Zhan Wang, Yaqin Yang. Conception: Huizi Ma. Design, Research project and Organization: Zhu Liu and Tao Feng. Review and Critique: Junhong Zhou and Yun Ling. Methodology: Zhonglue Chen and Kang Ren.

## FUNDING INFORMATION

This work was supported by the National Natural Science Foundation of China (Grant No. 81901833, Grant No. 82071422), Beijing Municipal Natural Science Foundation (7212031), and the Beijing Municipal Science & Technology Commission (Grant No. Z181100001718059).

## CONFLICT OF INTEREST STATEMENT

We declare that we have no financial and personal relationships with other people or organizations that can inappropriately influence our work. There is no professional or other personal interest of any nature or kind in any product, service, and/or company that could be construed as influencing the position presented in, or the review of, the submitted manuscript.

## Supporting information


Data S1.

## Data Availability

The data that support the findings of this study are available from the corresponding author upon reasonable request.
