# Supplementary material for: Machine‐learning model for the prediction of acute orthostatic hypotension after levodopa administration
Source: CNS Neurosci Ther. 2024 Mar 11;30(3):e14575. doi: 10.1111/cns.14575 (PMC10927600; doi:10.1111/cns.14575)
Supplement: Supplementary file 1 — Data S1. [file CNS-30-e14575-s001.docx]

**Supplemental section:**

**Methods:**

- - 1. **Study Protocol**

A comprehensive set of clinical features was assessed, including both motor and nonmotor symptoms. We used the following data:

(i) Demographics: age, sex, disease course, body mass index (BMI), daily levodopa equivalent unit (LEU), history of hypertension and diabetes mellitus.

(ii) Motor manifestations: All participants underwent LCT in accordance with published guidelines^28,29^. Motor response was evaluated by clinical rating according to the Movement Disorder Society-Unified Parkinson’s Disease Rating Scale (MDS-UPDRS)-Part III at baseline and at 1 h intervals following administration for up to 3 hours. For each patient, only the record of the best levodopa responsiveness was included in our study. Dose of levodopa/benserazide, BP change, score of MDS-UPDRS-III in off phase, tremor dominant (TD) and postural instability and gait disorders (PIGD) of motor phenotype according to MDS-UPDRS III ^30^, motor response, dystonia and other side effects during the tests were recorded. Levodopa responsiveness referred to MDS-UPDRS III total score change, $\%\Delta_{TotalScore}$:

$\%\Delta_{TotalScore}=(TotalScore_{baseline}-\min(TotalScore_{after-LCT})/TotalScore_{baseline}$, where $\min(TotalScore_{after-LCT})$ referred to the minimum MDS-UPDRS III total score within 3 hours after LCT.

(iii) Autonomic dysfunction: Patients consenting to participate were screened for symptoms of OH, including dizziness, faintness, and black spots, and symptoms that occurred while standing were recorded. STS was performed before drug administration and at 1, 2, and 3 hours after levodopa/benserazide was taken subsequently during the LCT.

(iv) Neuropsychological features: Mini-Mental State Examination (MMSE) and Montreal Cognitive Assessment (MoCA).

**2.2 Diagnostic Criteria and Definition**

- - 1. Orthostatic hypotension (OH) ﻿is ﻿a sustained reduction in SBP of at least 20 mm Hg or DBP of 10 mm Hg within 3 min of standing and may be symptomatic (i.e., ﻿dizziness, light headedness and syncope) or asymptomatic^6,32^.
    2. Supine hypertension was diagnosed according to the American Heart Association guidelines as follows: a systolic blood pressure (SBP) of ≥140 and/or diastolic blood pressure (DBP) of ≥ 90 mmHg^6^.
    3. Subtype: The ratio of the mean MDS-UPDRS tremor scores to the mean MDS-UPDRS PIGD scores was used to define TD patients (ratio ≥1.15), PIGD patients (ratio ≤0.9), and indeterminate patients (ratios >0.9 and <1.15)^30^.
    4. BP drop = supine BP – lowest standing BP; ***ΔDBP*** signifying DBP drop and ***ΔSBP*** signifying SBP drop;
    5. mean artery pressure (MAP) = (SBP + 2 ×DBP)/3 ^19^, ***Supine MAP*** signifying supine, ***Standing MAP*** signifying standing;

ΔMAP = ***Supine MAP* - *Standing MAP***.

2.2.6 Hypotension: a SBP of< 90mmHg and/or a DBP of <60 mmHg.

**Results:**

Supplemental figures:


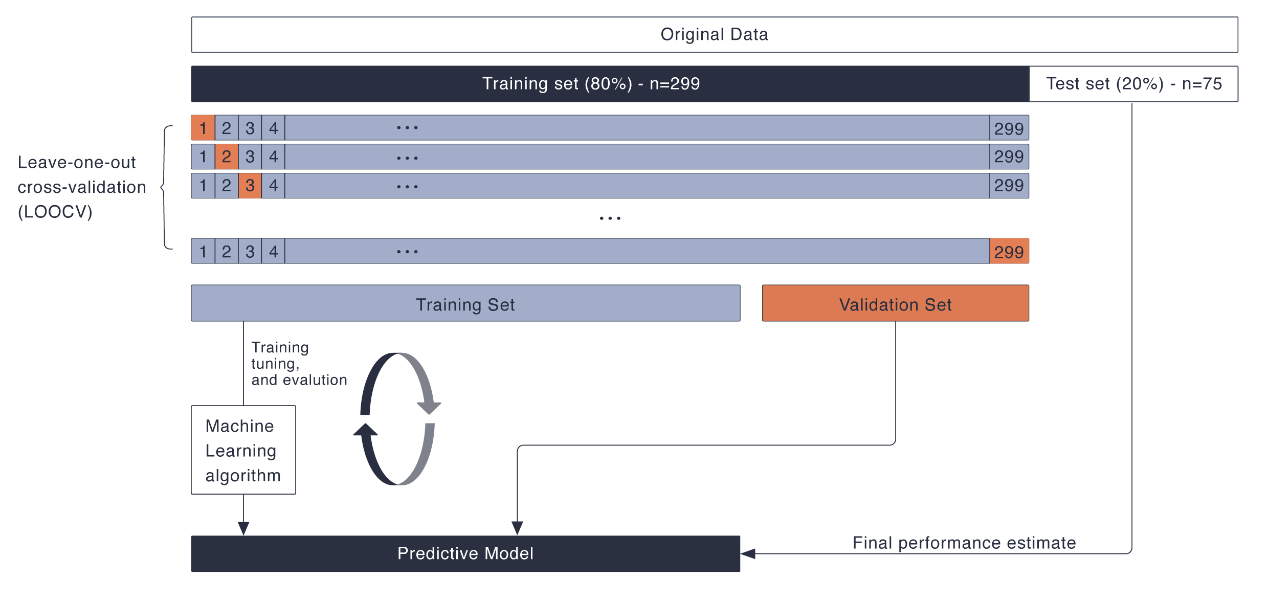


**Fig.S1 Model training and independent test data evaluation**

| 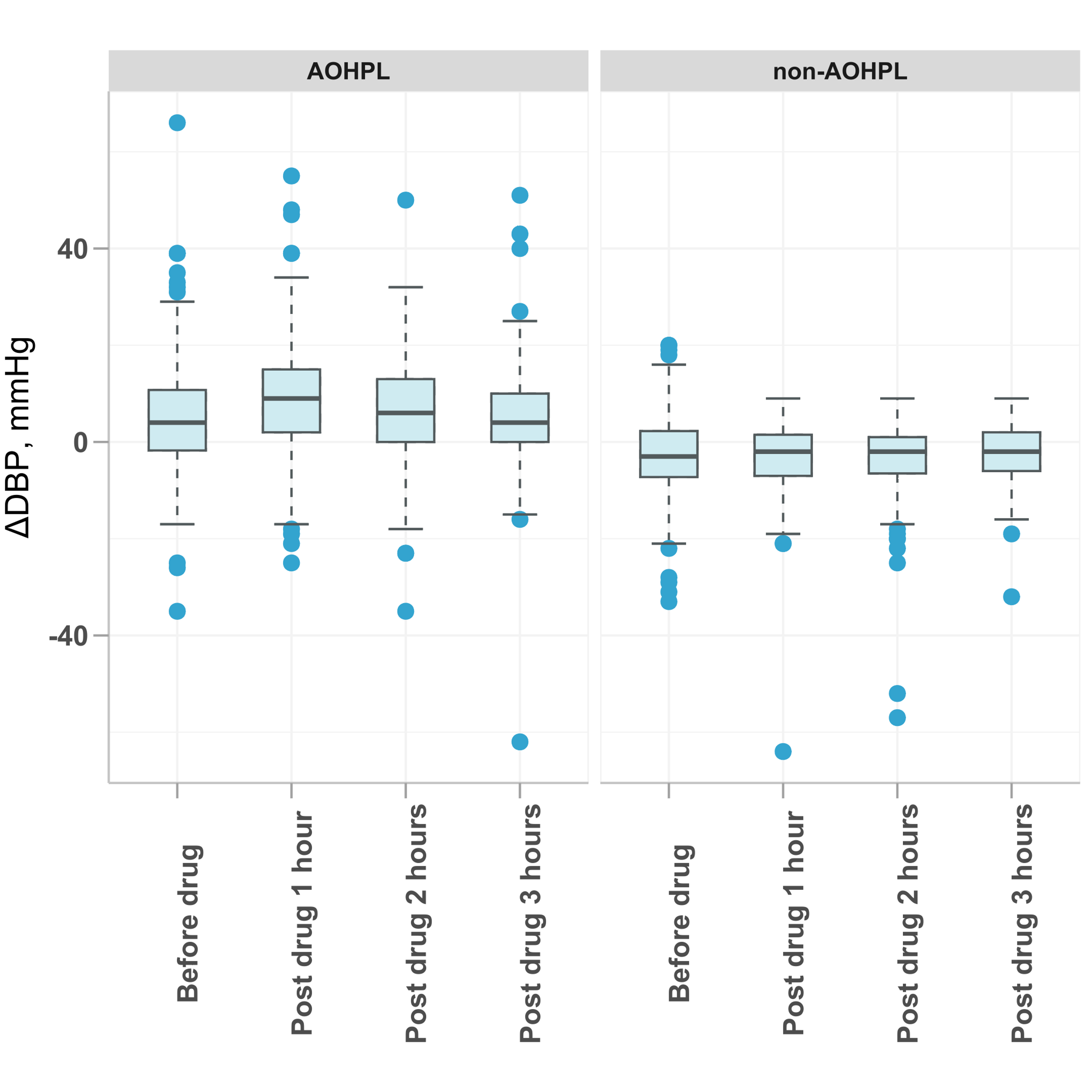 | 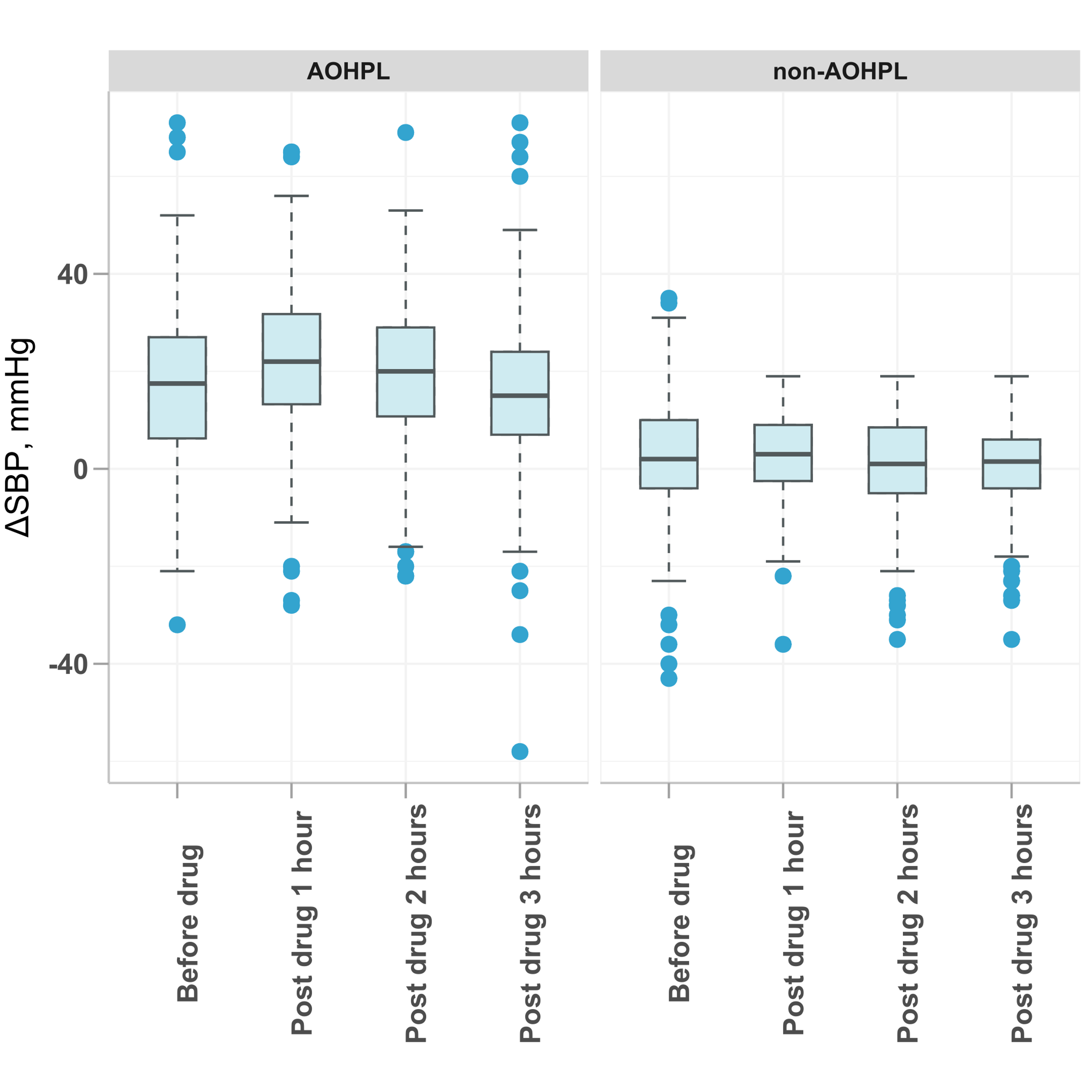 |
| --- | --- |
| 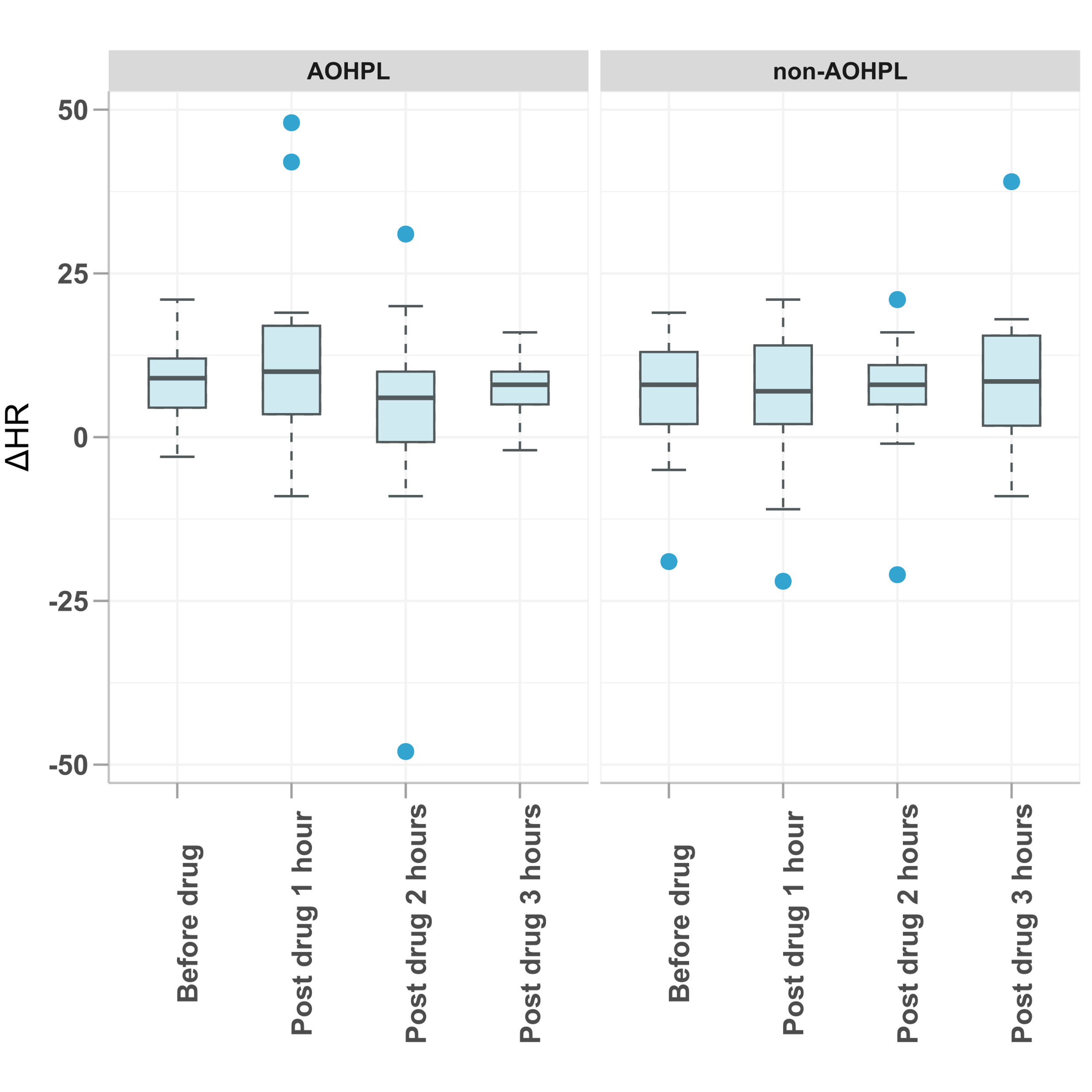 | 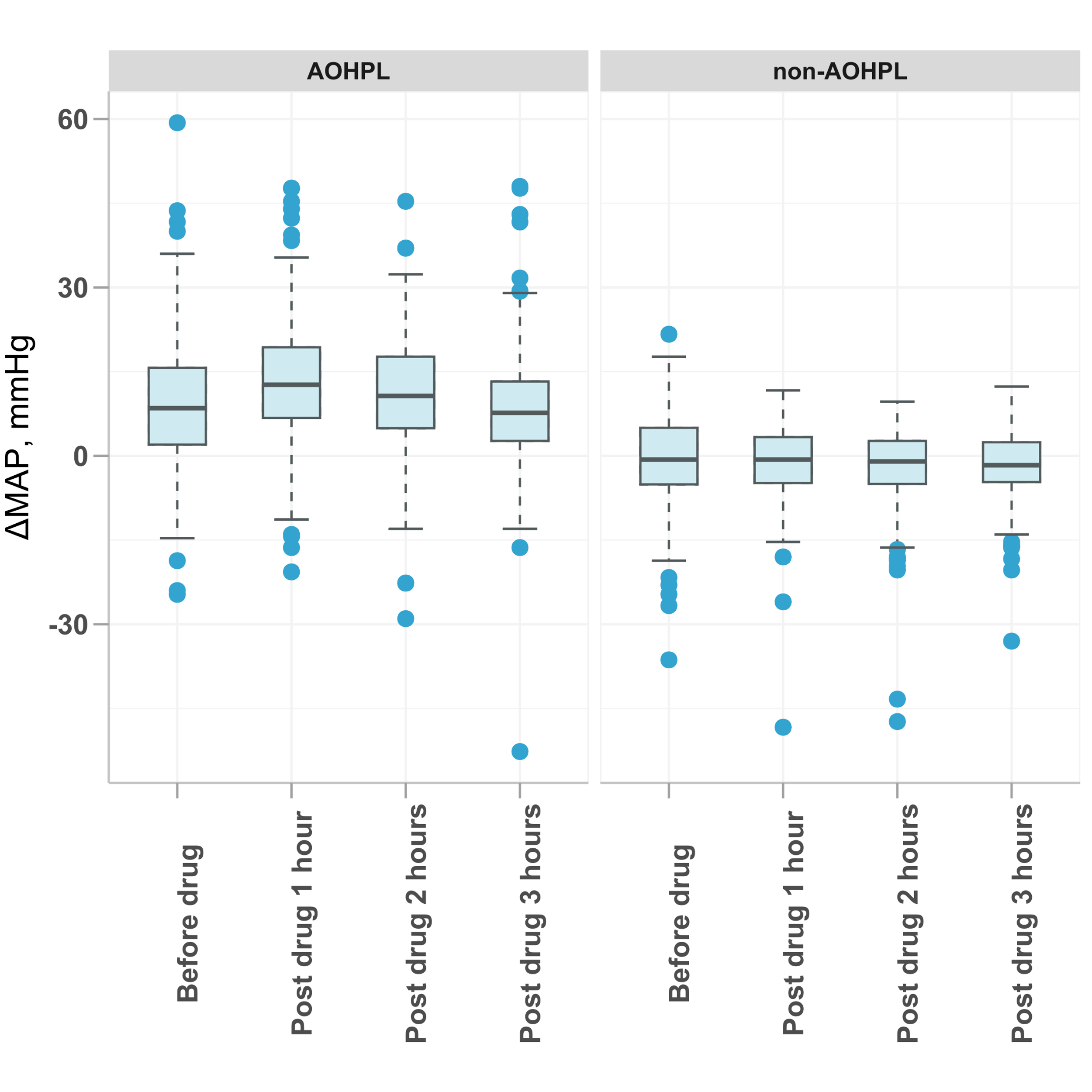 |

**Fig.S2 The trend in** Δ**SBP,** Δ**DBP,** Δ**HR, and** Δ**MAP of the subjects before and within 3 hours after medication.** Boxplot represents data as follows: 75^th^ quantile (Q3, top line of the box), median (central line of the box) and 25^th^ quantile (Q1, bottom line of the box), the bottom and top of the error bars indicate the "Minimum" (Q1-1.5*IQR) and "Maximum" (Q3+1.5* IQR), blue dots represent outliers (outside the range of Maximum and Minimum).

**
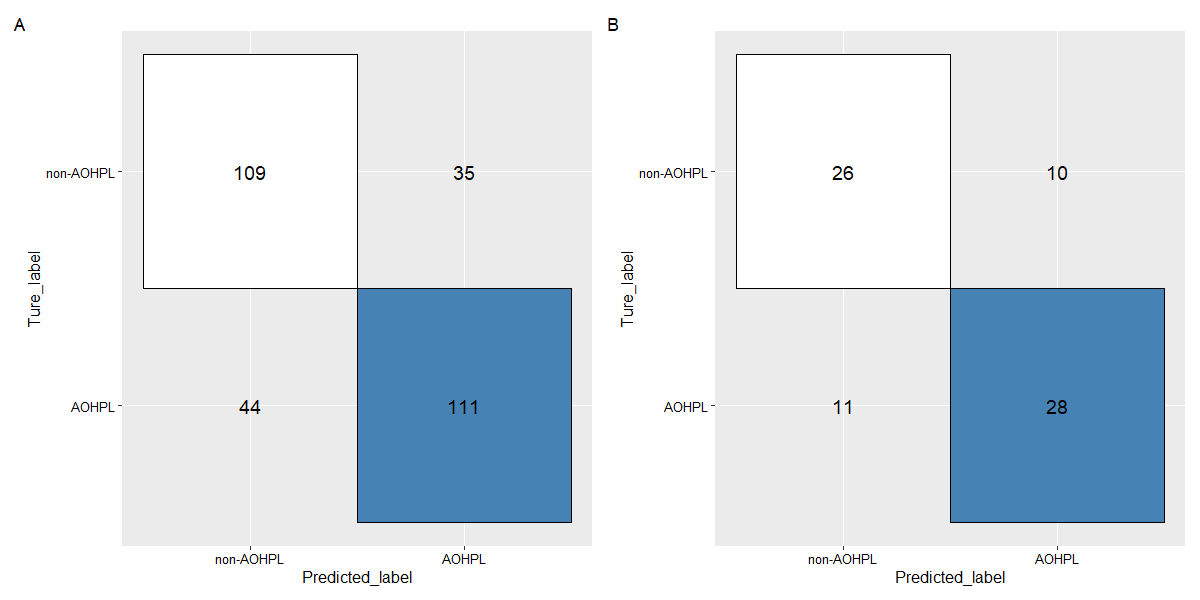
**

**Fig. S3 Confusion matrix tables of the predictive model on training data and test data. (A) Training data (B) Test data**

**Table S1 Demographic and clinical characteristics of participants**

| **Demographics & PD characteristics** | **Patients (n=497)** | **Missing, n** |
| --- | --- | --- |
| Age, years | 65.00 [58.00, 70.00] | 0 |
| Sex: Male, n (%) | 286 (57.7) | 1 |
| BMI, kg/m2 | 24.11 (3.50) | 26 |
| Use antihypertensive drugs, n (%) | 143 (28.8) | 0 |
| DM, n (%) | 69 (13.9) | 0 |
| Disease course, years | 6.00 [3.00, 9.00] | 1 |
| MDS-UPDRS III (OFF) | 39.00 [27.50, 52.00] | 2 |
| Subtype (%) |  | 1 |
| TD | 296 (59.7) |  |
| PIGD | 170 (34.3) |  |
| Indeterminate | 30 ( 6.0) |  |
| Dyskinesia, n (%) | 98 (19.9) | 4 |
| Dizziness, n (%) | 48 (10.2) | 27 |
| Supine hypertension, n (%) | 262 (52.8) | 1 |
| MMSE | 27.00 [24.00, 28.00] | 52 |
| MoCA | 21.00 [17.00, 25.00] | 53 |
| AOHPL, n (%) | 253 (51.5) | 6 |
| **Drugs** |  |  |
| LEU of overall antiparkinsonian drugs | 500.00 [187.50, 750.00] | 0 |
| LEU of Madopar and Sinemet | 256.25 [120.00, 440.00] | 0 |
| Dopamine receptor agonists, mg | 37.50 [0.00, 75.00] | 0 |
| MAO-B, mg | 0.00 [0.00, 0.00] | 0 |
| Benzhexol, mg | 0.00 [0.00, 0.00] | 0 |
| Amantadine | 0.00 [0.00, 100.00] | 0 |
| The dose of levodopa used in an LCT, mg | 125.00 [62.50, 187.50] | 0 |
| **Hemodynamics** |  |  |
| Supine SBP, mmHg | 136.00 [123.50, 150.00] | 2 |
| Standing SBP, mmHg | 126.00 [114.00, 140.00] | 4 |
| Supine DBP, mmHg | 82.00 [77.00, 90.00] | 2 |
| Standing DBP, mmHg | 82.00 [75.00, 90.00] | 4 |
| ΔSBP, mmHg | 8.00 [-1.00, 21.00] | 4 |
| ΔDBP, mmHg | 0.00 [-5.00, 7.00] | 4 |
| Supine MAP, mmHg | 100.67 [93.00, 109.33] | 2 |
| Standing MAP, mmHg | 97.33 [88.00, 105.00] | 4 |
| ΔMAP, mmHg | 3.33 [-3.00, 10.00] | 4 |
| Supine HR | 74.00 (11.64) | 445 |
| Standing HR | 82.31 (12.80) | 445 |
| ΔHR | 8.31 (8.10) | 445 |
| Supine HR 1h Post Drug | 73.00 [67.75, 80.00] | 445 |
| Standing HR 1h Post Drug | 83.38 (12.01) | 445 |
| ΔHR 1h Post Drug | 7.50 [3.00, 14.25] | 445 |
| Supine SBP 1h Post Drug, mmHg | 126.00 [113.00, 136.00] | 5 |
| Standing SBP 1h Post Drug, mmHg | 112.79 (20.73) | 8 |
| ΔSBP 1h Post Drug, mmHg | 10.00 [2.00, 24.00] | 8 |
| Supine DBP 1h Post Drug, mmHg | 77.00 [70.00, 85.00] | 5 |
| Standing DBP 1h Post Drug, mmHg | 74.00 [66.00, 82.00] | 8 |
| ΔDBP 1h Post Drug, mmHg | 2.00 [-3.00, 9.00] | 8 |
| Supine MAP 1h Post Drug, mmHg | 93.33 [85.67, 101.33] | 5 |
| Standing MAP 1h Post Drug, mmHg | 87.00 [78.33, 96.33] | 8 |
| ΔMAP 1h Post Drug, mmHg | 5.33 [-1.33, 13.33] | 8 |
| Supine HR 2h Post Drug | 76.50 [71.00, 85.75] | 447 |
| Standing HR 2h Post Drug | 87.20 (13.09) | 447 |
| ΔHR 2h Post Drug | 8.00 [4.25, 13.75] | 447 |
| Supine SBP 2h Post Drug, mmHg | 120.50 [110.00, 132.00] | 5 |
| Standing SBP 2h Post Drug, mmHg | 111.42 (20.86) | 11 |
| ΔSBP 2h Post Drug, mmHg | 8.50 [-1.00, 20.00] | 11 |
| Supine DBP 2h Post Drug, mmHg | 72.50 [65.00, 80.00] | 5 |
| Standing DBP 2h Post Drug, mmHg | 71.00 [63.00, 80.00] | 11 |
| ΔDBP 2h Post Drug, mmHg | 1.00 [-4.00, 7.00] | 11 |
| Supine MAP 2h Post Drug, mmHg | 89.00 [81.25, 96.00] | 5 |
| Standing MAP 2h Post Drug, mmHg | 84.80 (14.52) | 11 |
| ΔMAP 2h Post Drug, mmHg | 3.33 [-2.33, 10.67] | 11 |
| Supine HR 3h Post Drug | 79.93 (10.64) | 455 |
| Standing HR 3h Post Drug | 87.76 (11.62) | 456 |
| ΔHR 3h Post Drug | 7.00 [4.00, 14.00] | 456 |
| Supine SBP 3h Post Drug, mmHg | 123.00 [114.00, 134.75] | 87 |
| Standing SBP 3h Post Drug, mmHg | 115.26 (19.72) | 92 |
| ΔSBP 3h Post Drug, mmHg | 7.00 [-2.00, 17.00] | 92 |
| Supine DBP 3h Post Drug, mmHg | 74.00 [67.25, 80.00] | 87 |
| Standing DBP 3h Post Drug, mmHg | 73.00 [65.00, 80.00] | 92 |
| ΔDBP 3h Post Drug, mmHg | 1.00 [-3.00, 6.00] | 92 |
| Supine MAP 3h Post Drug, mmHg | 90.50 [84.00, 98.33] | 87 |
| Standing MAP 3h Post Drug, mmHg | 87.00 [78.67, 94.67] | 92 |
| ΔMAP 3h Post Drug, mmHg | 3.00 [-2.33, 9.00] | 92 |
| max ΔSBP Post Drug, mmHg | 16.00 [7.00, 30.00] | 6 |
| max ΔDBP Post Drug, mmHg | 6.00 [1.00, 13.00] | 6 |

Data are shown as the mean (SD) for normally distributed continuous variables, median (IQR) for nonnormally distributed continuous variables, and n (%) for categorical variables. PD: Parkinson’s disease; BMI: Body Mass Index; HT: hypertension; DM: diabetes mellitus; MDS‐UPDRS-III: Movement Disorders Society‐Unified Parkinson’s Disease Rating Scale part 3, PIGD: Postural instability and gait disorders; TD: tremor dominant; LEU: daily levodopa equivalent unit; MAO-B: monoamine oxidase-B; LCT: Acute levodopa challenge tests; AOHPL: post antiparkinsonian drug OH; MMSE: mini-mental state examination; MoCA: Montreal Cognitive Assessment; SBP: systolic blood pressure; DBP: diastolic blood pressure. HR: hear rate; Hypotension: a SBP of< 90mmHg and/or a DBP of <60 mmHg.

**Table S2 Hemodynamics characteristics of participants**

| **Hemodynamics** | **Overall** | **NON-AOHPL** | **AOHPL** | ***P*** |
| --- | --- | --- | --- | --- |
| **Before drug** |  |  |  |  |
| Standing DBP, mmHg | 81.00 [74.00, 88.75] | 83.00 [77.00, 90.00] | 79.00 [73.00, 88.00] | 0.001 |
| Standing HR | 81.40 (13.14) | 78.64 (13.24) | 86.00 (11.99) | 0.086 |
| Standing MAP, mmHg | 96.67 [87.33, 103.33] | 98.17 [90.08, 105.75] | 93.67 [86.08, 101.33] | <0.001 |
| Standing SBP, mmHg | 124.00 [114.00, 138.00] | 128.00 [115.00, 141.00] | 121.00 [109.00, 134.75] | 0.001 |
| Supine DBP, mmHg | 82.00 [77.00, 89.75] | 80.00 [75.00, 87.00] | 84.00 [78.00, 90.75] | <0.001 |
| Supine HR | 74.22 (12.10) | 72.44 (11.94) | 77.20 (12.19) | 0.233 |
| Supine MAP, mmHg | 101.00 [93.33, 108.25] | 98.00 [91.25, 105.75] | 103.00 [95.67, 110.25] | <0.001 |
| Supine SBP, mmHg | 136.61 (18.77) | 132.51 (17.77) | 140.43 (18.91) | <0.001 |
| ΔDBP, mmHg | 0.00 [-4.00, 7.00] | -3.00 [-7.25, 2.25] | 4.00 [-1.75, 10.75] | <0.001 |
| ΔHR | 7.18 (8.04) | 6.20 (8.85) | 8.80 (6.44) | 0.329 |
| ΔMAP, mmHg | 3.67 [-3.00, 10.00] | -0.67 [-5.08, 5.00] | 8.50 [2.00, 15.67] | <0.001 |
| ΔSBP, mmHg | 9.00 [-0.75, 22.00] | 2.00 [-4.00, 10.00] | 17.50 [6.25, 27.00] | <0.001 |
| **1h Post-drug** |  | | |  |
| Standing DBP, mmHg | 74.00 [65.00, 81.00] | 78.00 [71.00, 86.50] | 69.00 [61.00, 76.00] | <0.001 |
| Standing HR | 83.15 (12.22) | 79.52 (11.26) | 89.20 (11.66) | 0.013 |
| Standing MAP, mmHg | 86.67 [77.00, 95.33] | 91.00 [85.00, 101.33] | 81.33 [72.33, 89.25] | <0.001 |
| Standing SBP, mmHg | 112.22 (20.65) | 119.81 (18.48) | 105.21 (20.10) | <0.001 |
| Supine DBP, mmHg | 76.00 [70.00, 84.00] | 75.00 [69.00, 82.00] | 77.00 [71.25, 85.00] | 0.063 |
| Supine HR | 74.40 (11.03) | 73.48 (12.15) | 75.93 (9.04) | 0.503 |
| Supine MAP, mmHg | 92.67 [85.67, 100.67] | 90.33 [84.00, 100.00] | 94.33 [88.08, 101.58] | 0.010 |
| Supine SBP, mmHg | 125.00 [112.00, 136.00] | 122.00 [110.00, 133.00] | 127.00 [116.25, 136.00] | 0.005 |
| ΔDBP, mmHg | 2.00 [-3.00, 9.00] | -2.00 [-7.00, 1.50] | 9.00 [2.00, 15.00] | <0.001 |
| ΔHR | 7.50 [3.00, 14.25] | 7.00 [2.00, 14.00] | 10.00 [3.50, 17.00] | 0.229 |
| ΔMAP, mmHg | 5.33 [-1.33, 13.00] | -0.67 [-4.83, 3.33] | 12.67 [6.75, 19.33] | <0.001 |
| ΔSBP, mmHg | 10.00 [2.00, 23.00] | 3.00 [-2.50, 9.00] | 22.00 [13.25, 31.75] | <0.001 |
| **2h Post-drug** |  | | |  |
| Standing DBP, mmHg | 71.00 [62.00, 79.00] | 75.00 [69.50, 82.50] | 65.00 [58.00, 74.00] | <0.001 |
| Standing HR | 87.15 (13.25) | 86.36 (12.79) | 88.57 (14.41) | 0.623 |
| Standing MAP, mmHg | 84.32 (14.54) | 90.54 (12.29) | 78.52 (14.10) | <0.001 |
| Standing SBP, mmHg | 110.82 (21.05) | 119.08 (17.61) | 103.11 (21.10) | <0.001 |
| Supine DBP, mmHg | 72.00 [65.00, 80.00] | 72.00 [66.00, 79.00] | 72.50 [65.00, 80.00] | 0.965 |
| Supine HR | 78.00 [71.00, 88.00] | 78.00 [72.00, 82.00] | 81.00 [69.25, 91.50] | 0.714 |
| Supine MAP, mmHg | 89.00 [81.67, 96.00] | 88.67 [81.17, 95.00] | 89.33 [81.67, 96.67] | 0.559 |
| Supine SBP, mmHg | 120.00 [110.00, 130.00] | 120.00 [110.00, 129.00] | 121.00 [110.25, 132.75] | 0.185 |
| ΔDBP, mmHg | 1.00 [-4.00, 7.00] | -2.00 [-6.50, 1.00] | 6.00 [0.00, 13.00] | <0.001 |
| ΔHR | 7.00 [3.50, 11.00] | 8.00 [5.00, 11.00] | 6.00 [-0.75, 10.00] | 0.472 |
| ΔMAP, mmHg | 4.00 [-2.00, 11.00] | -1.00 [-5.00, 2.67] | 10.67 [4.92, 17.67] | <0.001 |
| ΔSBP, mmHg | 9.94 (15.90) | 0.58 (10.23) | 18.66 (15.28) | <0.001 |
| **3h Post-drug** |  | | |  |
| Standing DBP, mmHg | 72.50 [65.00, 80.00] | 74.50 [69.75, 82.00] | 69.00 [62.00, 77.75] | <0.001 |
| Standing HR | 87.12 (11.72) | 87.60 (13.27) | 86.38 (9.31) | 0.776 |
| Standing MAP, mmHg | 86.67 [78.67, 94.25] | 90.33 [83.58, 96.08] | 82.83 [74.67, 90.92] | <0.001 |
| Standing SBP, mmHg | 114.46 (20.02) | 120.44 (16.83) | 109.54 (21.11) | <0.001 |
| Supine DBP, mmHg | 73.00 [67.00, 80.00] | 73.00 [67.00, 79.00] | 73.00 [68.00, 80.00] | 0.217 |
| Supine HR | 79.09 (10.42) | 79.19 (11.06) | 78.92 (9.74) | 0.943 |
| Supine MAP, mmHg | 90.00 [83.75, 96.33] | 89.00 [82.17, 95.33] | 91.67 [84.83, 98.67] | 0.059 |
| Supine SBP, mmHg | 123.00 [114.00, 133.00] | 121.00 [112.00, 131.00] | 124.00 [115.00, 135.00] | 0.039 |
| ΔDBP, mmHg | 1.00 [-3.00, 6.00] | -2.00 [-6.00, 2.00] | 4.00 [0.00, 10.00] | <0.001 |
| ΔHR | 8.00 [4.00, 14.00] | 8.50 [1.75, 15.50] | 8.00 [5.00, 10.00] | 0.580 |
| ΔMAP, mmHg | 3.00 [-2.33, 9.25] | -1.67 [-4.67, 2.42] | 7.67 [2.67, 13.25] | <0.001 |
| ΔSBP, mmHg | 7.00 [-2.00, 17.00] | 1.50 [-4.00, 6.00] | 15.00 [7.00, 24.00] | <0.001 |

Data are shown as the mean (SD) for normally distributed continuous variables, median (IQR) for nonnormally distributed continuous variables, and n (%) for categorical variables.**Table S3 Contingency table for OH before and after medication**

|  | **pre-drug OH** | **Non pre-drug OH** |
| --- | --- | --- |
| **AOHPL** | 95 | 99 |
| **non-AOHPL** | 27 | 153 |

**Table S4 Comparison of main features for non-AOHPL between training data and test data**

|  | **Training set** | **Test set** | **p** |
| --- | --- | --- | --- |
| N | 144 | 36 |  |
| Age, years | 62.50 [56.00, 69.00] | 62.50 [56.00, 68.75] | 0.791 |
| Sex: Male, n (%) | 88 (61.1) | 19 (52.8) | 0.471 |
| BMI, kg/m2 | 24.22 (3.83) | 24.46 (3.89) | 0.733 |
| Use antihypertensive drugs, n (%) | 34 (23.6) | 12 (33.3) | 0.326 |
| DM, n (%) | 13 (9.0) | 6 (16.7) | 0.303 |
| Disease course, years | 6.00 [3.00, 8.00] | 5.00 [2.75, 8.00] | 0.658 |
| MDS-UPDRS III (OFF) | 37.00 [26.00, 46.25] | 39.50 [26.50, 53.25] | 0.393 |
| OH, pre_drug | 22 (15.3) | 5 (13.9) | 1.000 |
| Subtype (%) |  |  | 0.400 |
| TD | 80 (55.6) | 23 (63.9) |  |
| PIGD | 51 (35.4) | 12 (33.3) |  |
| Indeterminate | 13 (9.0) | 1 (2.8) |  |
| Dyskinesia, n (%) | 31 (21.5) | 3 (8.3) | 0.116 |
| Dizziness, n (%) | 10 (6.9) | 1 (2.8) | 0.586 |
| Supine hypertension, n (%) | 58 (40.3) | 18 (50.0) | 0.386 |
| MMSE | 27.00 [24.00, 29.00] | 26.00 [25.00, 29.00] | 0.557 |
| MoCA | 22.00 [18.00, 25.00] | 22.50 [19.00, 25.25] | 0.686 |
| **Drug** |  |  |  |
| LEU of overall antiparkinsonian drugs | 475.00 [268.75, 703.12] | 325.00 [75.00, 643.75] | 0.132 |
| LEU of Madopar and Sinemet | 288.12 [120.00, 410.62] | 220.00 [0.00, 302.19] | **0.048** |
| Dopamine receptor agonists, mg | 37.50 [0.00, 75.00] | 25.00 [0.00, 81.25] | 0.560 |
| MAO-B, mg | 0.00 [0.00, 0.00] | 0.00 [0.00, 0.00] | 0.544 |
| Benzhexol, mg | 0.00 [0.00, 0.00] | 0.00 [0.00, 0.00] | 0.126 |
| Amantadine | 0.00 [0.00, 100.00] | 0.00 [0.00, 125.00] | 0.555 |
| The dose of levodopa used in an LCT, mg | 125.00 [62.50, 187.50] | 125.00 [62.50, 140.62] | 0.091 |
| **Hemodynamics** |  |  |  |
| Supine SBP, mmHg | 131.93 (17.67) | 134.81 (18.25) | 0.387 |
| Standing SBP, mmHg | 127.50 [115.75, 139.25] | 132.00 [114.75, 142.75] | 0.428 |
| Supine DBP, mmHg | 80.00 [74.75, 87.00] | 80.00 [75.00, 87.25] | 0.923 |
| Standing DBP, mmHg | 82.50 [77.00, 89.00] | 85.00 [80.50, 93.25] | 0.137 |
| ΔSBP, mmHg | 3.00 [-2.00, 10.00] | -1.50 [-9.00, 9.50] | 0.179 |
| ΔDBP, mmHg | -3.00 [-6.25, 3.00] | -4.00 [-8.00, -1.00] | 0.088 |
| Supine MAP, mmHg | 98.00 [91.00, 104.83] | 96.17 [91.58, 106.67] | 0.734 |
| Standing MAP, mmHg | 97.67 [89.17, 104.42] | 101.00 [91.08, 110.50] | 0.176 |
| ΔMAP, mmHg | 0.00 [-4.17, 5.08] | -3.67 [-8.42, 4.75] | 0.110 |

Data are shown as the mean (SD) for normally distributed continuous variables, median (IQR) for nonnormally distributed continuous variables, and n (%) for categorical variables. BMI: Body Mass Index; HT: hypertension; DM: diabetes mellitus; MDS‐UPDRS-III: Movement Disorders Society‐Unified Parkinson's Disease Rating Scale part; PIGD: Postural instability and gait disorders; TD: tremor dominant; LEU: daily levodopa equivalent unit; MAO-B: monoamine oxidase-B; LCT: Acute levodopa challenge tests; AOHPL: post antiparkinsonian drug OH; MMSE: mini-mental state examination; MoCA: Montreal Cognitive Assessment.

**Table S5 Comparison of main features for AOHPL between training data and test data**

|  | **Training set** | **Test set** | **p** |
| --- | --- | --- | --- |
| N | 155 | 39 |  |
| Age, years | 66.00 [61.00, 71.00] | 65.00 [62.00, 68.50] | 0.383 |
| Sex: Male, n (%) | 95 (61.3) | 22 (56.4) | 0.709 |
| BMI, kg/m2 | 23.97 (3.32) | 24.46 (3.10) | 0.398 |
| Use antihypertensive drugs, n (%) | 44 (28.4) | 11 (28.2) | 1.000 |
| DM, n (%) | 28 (18.1) | 6 (15.4) | 0.875 |
| Disease course, years | 6.00 [4.00, 9.50] | 6.00 [3.50, 10.00] | 0.831 |
| MDS-UPDRS III (OFF) | 39.00 [28.50, 54.00] | 44.00 [32.00, 53.00] | 0.557 |
| pre-drug OH | 75 (48.4) | 20 (51.3) | 0.885 |
| Subtype (%) |  |  | 0.588 |
| TD | 95 (61.3) | 21 (53.8) |  |
| PIGD | 53 (34.2) | 15 (38.5) |  |
| Indeterminate | 7 (4.5) | 3 (7.7) |  |
| Dyskinesia, n (%) | 41 (26.5) | 5 (12.8) | 0.114 |
| Dizziness, n (%) | 23 (14.8) | 6 (15.4) | 1.000 |
| Supine hypertension, n (%) | 98 (63.2) | 23 (59.0) | 0.760 |
| MMSE | 27.00 [23.00, 28.50] | 26.00 [23.00, 27.50] | 0.269 |
| MoCA | 21.00 [17.50, 24.00] | 21.00 [15.50, 24.00] | 0.590 |
| **Drug** |  |  |  |
| LEU of overall antiparkinsonian drugs | 540.50 [268.75, 756.25] | 537.50 [200.00, 799.50] | 0.708 |
| LEU of Madopar and Sinemet | 320.00 [160.00, 480.00] | 240.00 [140.00, 480.00] | 0.898 |
| Dopamine receptor agonists, mg | 37.50 [0.00, 100.00] | 0.00 [0.00, 87.50] | 0.156 |
| MAO-B, mg | 0.00 [0.00, 0.00] | 0.00 [0.00, 0.00] | 0.242 |
| Benzhexol, mg | 0.00 [0.00, 0.00] | 0.00 [0.00, 0.00] | 0.697 |
| Amantadine | 0.00 [0.00, 100.00] | 0.00 [0.00, 50.00] | 0.815 |
| The dose of levodopa used in an LCT, mg | 125.00 [62.50, 187.50] | 125.00 [93.75, 218.75] | 0.834 |
| **Hemodynamics** |  |  |  |
| Supine SBP, mmHg | 140.14 (19.40) | 141.56 (17.03) | 0.676 |
| Standing SBP, mmHg | 121.00 [108.00, 133.00] | 127.00 [115.00, 139.00] | 0.177 |
| Supine DBP, mmHg | 84.00 [78.00, 90.00] | 85.00 [79.00, 92.50] | 0.496 |
| Standing DBP, mmHg | 79.00 [73.00, 88.00] | 80.00 [73.50, 84.50] | 0.933 |
| ΔSBP, mmHg | 18.00 [5.50, 26.00] | 17.00 [9.00, 27.50] | 0.844 |
| ΔDBP, mmHg | 3.00 [-1.50, 9.50] | 5.00 [-2.00, 13.50] | 0.482 |
| Supine MAP, mmHg | 102.33 [95.67, 109.17] | 103.33 [95.33, 112.83] | 0.438 |
| Standing MAP, mmHg | 93.00 [85.50, 101.33] | 96.33 [87.83, 100.67] | 0.431 |
| ΔMAP, mmHg | 7.33 [2.17, 14.83] | 10.33 [-0.50, 17.33] | 0.446 |

Data are shown as the mean (SD) for normally distributed continuous variables, median (IQR) for nonnormally distributed continuous variables, and n (%) for categorical variables. BMI: Body Mass Index, HT: hypertension, DM: diabetes mellitus, MDS‐UPDRS-III: Movement Disorders Society‐Unified Parkinson's Disease Rating Scale part 3, PIGD: Postural instability and gait disorders, TD: tremor dominant, LEU: daily levodopa equivalent unit, MAO-B: monoamine oxidase-B, LCT: Acute levodopa challenge tests, AOHPL: post antiparkinsonian drug OH, MMSE: mini-mental state examination, MoCA: Montreal Cognitive Assessment.

**Table S6 Model performance distribution with 10 times randomly split into training and test dataset**

| **Performance indices** | **Test data** | **Training data** |
| --- | --- | --- |
| AUC_ROC | 0.72 [0.67, 0.73] | 0.75 [0.74, 0.77] |
| Accuracy | 0.61 [0.56, 0.63] | 0.73 [0.71, 0.74] |
| Kappa | 0.21 [0.10, 0.26] | 0.45 [0.43, 0.47] |
| Sensitivity | 0.81±0.18 | 0.69±0.04 |
| Specificity | 0.31 [0.22, 0.56] | 0.77 [0.76, 0.78] |
| PPV | 0.58 [0.55, 0.62] | 0.76 [0.76, 0.78] |
| NPV | 0.70±0.15 | 0.70±0.02 |

**Table S7 The first three variables with most contribution in independent test data**

|  |  |  | **1st variable** | | **2nd variable** | | **3rd variable** | |
| --- | --- | --- | --- | --- | --- | --- | --- | --- |
| **ID** | **Prob of**  **AOHPL** | **Class**  **predicted** | **Features**  **(value)** | **Impact** | **Features (value)** | **Impact** | **Features (value)** | **Impact** |
| 60 | 0.41 | 0 | ΔSBP (-9) | -0.124 | Age (46) | -0.072 | ΔDBP (12) | 0.065 |
| 61 | 0.54 | 1 | ΔSBP (-3) | -0.119 | Supine hypertension (1) | 0.053 | Standing MAP (109.33) | -0.051 |
| 70 | 0.31 | 0 | ΔSBP (8) | -0.118 | Age (59) | -0.058 | Supine hypertension (0) | -0.053 |

**Table S8 Most frequent in the top three variables in independent test data**

| **order** | **ΔSBP** | **ΔDBP** | **Standing MAP** |
| --- | --- | --- | --- |
| 1st | 50 | 24 | 0 |
| 2nd | 23 | 27 | 9 |
| 3rd | 2 | 10 | 17 |
| Total | 75 | 61 | 26 |
